# Laterally positioned flap to treat deep isolated gingival recession in a rotated tooth. A case report

**DOI:** 10.21142/2523-2754-1401-2026-279

**Published:** 2025-12-28

**Authors:** Evelyn Salazar, Andrea Vergara-Buenaventura

**Affiliations:** 1 Department of Periodontics, Faculty of Health Sciences, Universidad Peruana de Ciencias Aplicadas. Lima, Peru. E202310936@upc.edu.pe peandver@upc.edu.pe Universidad Peruana de Ciencias Aplicadas Department of Periodontics Faculty of Health Sciences Universidad Peruana de Ciencias Aplicadas Lima Peru E202310936@upc.edu.pe peandver@upc.edu.pe

**Keywords:** gingival recession, laterally positioned flap, orthodontics, periodontium, phenotype, colgajo lateralmente posicionado, fenotipo, ortodoncia, periodonto, recesión gingival

## Abstract

**Background:**

Complete root coverage (CRC) is the primary goal in the treatment of gingival recession defects. However, anatomical factors such as thin periodontal phenotype, limited keratinized tissue width, mucosal thickness, and tooth position can influence treatment prognosis. The laterally moved flap technique is recommended when keratinized tissue is absent apical to the recession defect, especially in malpositioned teeth.

**Methods:**

A 36-year-old woman presented with a Miller Class II (Cairo Recession Type 2) gingival recession defect on the buccal surface of a rotated tooth #24, characterized by absence of keratinized tissue and a thin gingival phenotype. A laterally moved coronally advanced flap combined with a connective tissue graft was performed to increase keratinized tissue and mucosal thickness prior to orthodontic treatment.

**Results:**

At 6 months post-surgery, the patient showed stable clinical conditions with 60% root coverage, significant gain of keratinized tissue (3 mm), and increased gingival thickness. Both donor and recipient sites demonstrated satisfactory healing without signs of inflammation.

**Conclusion:**

The combined use of a laterally moved coronally advanced flap with connective tissue graft is an effective mucogingival approach for managing deep gingival recession in rotated teeth with thin phenotypes. This technique enhances tissue thickness and keratinized tissue width, improving periodontal stability prior to orthodontic intervention

## RESUMEN

**Antecedentes:**

La cobertura radicular completa es el objetivo principal del tratamiento de los defectos por recesión gingival (RG). Sin embargo, factores anatómicos como un fenotipo periodontal delgado, el escaso ancho del tejido queratinizado, el grosor de la mucosa y la posición dentaria pueden influir en el pronóstico del tratamiento. La técnica del colgajo desplazado lateralmente está recomendada cuando no hay tejido queratinizado apical al defecto de RG, especialmente en dientes mal posicionados.

**Materiales y métodos:**

Una paciente de 36 años presentó una RG clase II de Miller (RG tipo 2, según Cairo), caracterizada por la ausencia de tejido queratinizado y un fenotipo gingival delgado, en la superficie vestibular de un diente rotado (#24). Se realizó un colgajo desplazado lateralmente con avance coronal con un injerto de tejido conectivo, con el objetivo de aumentar el tejido queratinizado y el grosor mucoso antes del tratamiento ortodóntico.

**Resultados:**

A los 6 meses posoperatorios, la paciente mostró condiciones clínicas estables, con una cobertura radicular del 60%, una ganancia significativa de tejido queratinizado (3 mm) e incremento del grosor gingival. Tanto el sitio donante como el receptor presentaron una cicatrización satisfactoria sin signos de inflamación.

**Conclusión:**

El uso combinado de un colgajo desplazado lateralmente con avance coronal e injerto de tejido conectivo constituye un enfoque mucogingival eficaz para el manejo de recesiones gingivales profundas en dientes rotados con fenotipos delgados. Esta técnica mejora el grosor tisular y la anchura del tejido queratinizado, lo que favorece la estabilidad periodontal previa al tratamiento ortodóntico.

## INTRODUCTION

Gingival recession (GR) is defined as the apical migration of the gingival margin beyond the cemento-enamel junction (CEJ^) (^^1^. It is a mucogingival condition with significant quality of life implications, such as root caries and dentin hypersensitivity ^2^^,^^3^. 

GR is a multifactorial condition associated with various clinical and anatomical conditions ^3^. When assessing the GR for surgical intervention, evaluation of these anatomical conditions including periodontal phenotype, keratinized tissue(KT) width, mucosal thickness (MT) should be considered ^3^^,^^4^. Gingival augmentation procedures include flaps and grafts with the primary goal of achieving complete root coverage (CRC) ^5^. However, the presence of severe dental malposition, rotation, and/or extrusion can negatively affect the prognosis of CRC ^(5, 6)^.

Tooth rotation (TR) is a dental anomaly with a reported prevalence between 5% and 30% ^7^. It is characterized by the misalignment of a tooth along its long axis, resulting in a deviation from its normal position within the dental arch ^8^. Although its exact etiology is unknown, it may originate from disturbances in dental development, genetic predisposition, or environmental and/or occlusal factors ^9^. Rotated teeth typically exhibit a thinner or more apically positioned buccal bone plate compared to adjacent teeth ^6^. Furthermore, TR alters the position of the interdental contact point relative to the CEJ, which affects the height of the papilla and, consequently, the predictability of CRC ^6^^,^^10^. As a result, the CEJ should not be used as the reference point for root coverage in such cases ^6^.

Orthodontic treatment (OT) is often recommended prior to periodontal plastic surgery in cases involving malpositioned teeth, as dental malposition may exacerbate GR ^6^. However, tooth movement during OT induces changes in the periodontal tissues, especially in rotated teeth with a thin phenotype ^11^^,^^12^. It has been shown that GR may initiate or progress during OT depending on the direction of tooth movement ^3^^,^^12^. When orthodontic movement is required in a tooth already affected by GR, the decision-making process should be based on the thickness of the surrounding tissues ^13^. If there is a high risk of additional GR or the patient exhibits a thin periodontal phenotype, mucogingival surgery should be performed before OT ^12^. Periodontal preparation is therefore essential to support tooth movement and prevent further attachment loss ^14^^,^^15^.

Phenotypic modification leads to both clinical and histological changes, including an increase in KT width and thickness, typically achieved through soft tissue grafts ^16^. Histologically, this results in a thicker epithelial layer and a greater number and density of collagen fibers in the lamina propria ^16^^,^^17^.

This case report describes the mucogingival management of a rotated premolar with a thin gingival phenotype using a laterally positioned coronally advanced flap and a connective tissue graft, performed prior to orthodontic treatment.

## CASE REPORT

The present case report followed the recommendations of the Case Report guidelines ^18^ and presents patient consent for publication.

### Clinical presentation

**Fig. 1:**
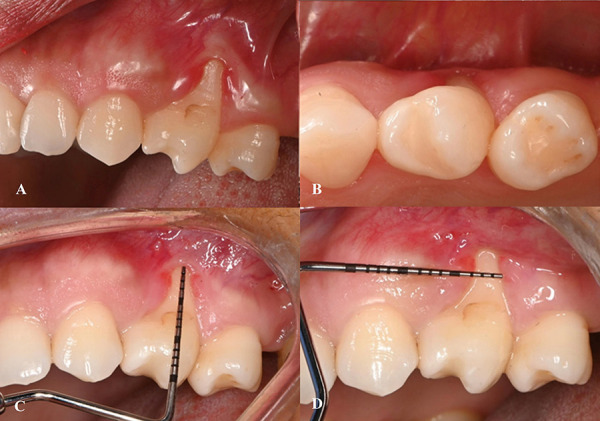
Clinical preoperative images of the deep, isolated gingival recession on a rotated mandibular premolar. A) Buccal view showing a deep gingival recession defect with absence of keratinized tissue and a thin gingival phenotype. B) Occlusal view highlighting the malposition of tooth #24 and the insufficient soft tissue coverage. C) Measurement of the gingival recession defect, confirming clinical attachment loss and lack of keratinized tissue. D) Measurement of the width of the remaining keratinized tissue using a periodontal probe, further confirming the thin biotype and soft tissue deficiency.

**Fig. 2:**
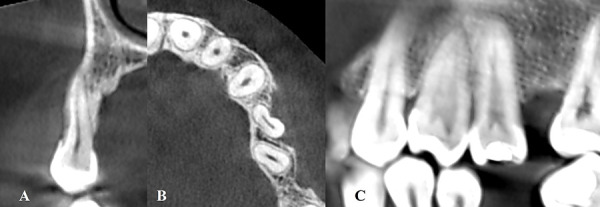
CBCT images of the affected area, highlighting the anatomical conditions relevant to the gingival recession in tooth #24. A) Sagittal CBCT slice showing buccal bone dehiscence and root prominence of the rotated mandibular premolar. B) Axial CBCT view displaying the rotation and vestibular displacement of tooth #24, confirming its malposition within the alveolar bone. C) Coronal CBCT section revealing reduced buccal bone thickness and fenestration in the region of interest, indicating a compromised periodontal architecture.

A 36-year-old woman with a non-contributory medical history presented to the Department of Periodontics at Universidad Peruana de Ciencias Aplicadas for evaluation of gingival recession (GR) at tooth #24 prior to OT. Clinical examination revealed distal rotation (gyroversion) of tooth #24, with a deep buccal GR measuring 7 mm, a thin gingival phenotype, absence of keratinized tissue, and a shallow vestibule (Fig. 1). Cone Beam Computed Tomography analysis assessment showed loss of vestibular table and distal interdental bone in tooth #24 (Fig. 2). The patient reported dentin hypersensitivity but did not exhibit any discomfort upon percussion. Patient was diagnosed with gingivitis associated with dental biofilm on a reduced periodontium ^18^. The GR at tooth #24 was classified as Miller Class II and Cairo Recession Type 2 (RT2) ^3^. Notably, the adjacent tooth (#23) exhibited sufficient keratinized tissue (>3 mm), making it suitable as a donor site.

### Case management

Ten days prior to surgery, supragingival prophylaxis was performed, and the patient received personalized oral hygiene instructions. After obtaining signed informed consent, local anesthesia was administered using 2% lidocaine hydrochloride with 1:80,000 epinephrine. A laterally moved coronally advanced flap combined with a connective tissue graft (CTG), following the technique described by Zucchelli et al. ^19^, was planned.

The recession depth was measured, and an oblique, submarginal, partial-thickness incision was made in the interdental area between teeth #23 and #24, extending 3 mm beyond the mucogingival junction. The marginal gingiva and papilla at the donor site were preserved. Muscle release was achieved by dissecting the underlying muscle and connective tissue fibers apical to the mucogingival junction, thereby reducing muscle tension and enabling tension-free coronal displacement of the flap.

**Fig. 3:**
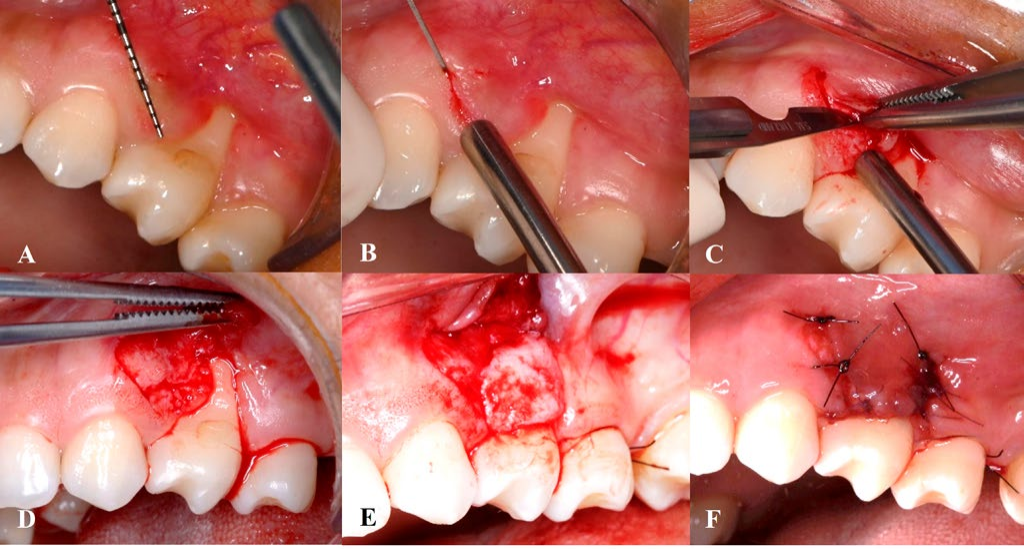
Surgical sequence of the laterally positioned flap combined with connective tissue garfting. A) Surgical planning of the recipient area and determining the incision line for lateral flap design. B) Incision performed in the interdental area between teeth #23 and #24 preserving the marginal gingiva and papilla. C) Partial-thickness incision. D) Partial-thickness flap elevation. E) Connective tissue graft placement on the recession defect prior to lateral flap repositioning. F) Suturing of the laterally positioned flap over the grafted area using interrupted sutures, ensuring full coverage and stability.

A sterile paper template was used to replicate the exact dimensions of the recipient bed. The template was then positioned over the palatal donor site to guide the harvesting of the CTG. A free gingival graft was harvested from the palate and de-epithelialized extraorally. The exposed root surface was mechanically debrided with Gracey curettes to remove bacterial contaminants. The CTG was then positioned and secured at the recipient site using simple interrupted sutures with 6/0 polyglycolic acid. The laterally displaced flap was subsequently stabilized at the base of the papilla with simple interrupted sutures using 5/0 nylon (Fig. 3).

**Fig. 4:**
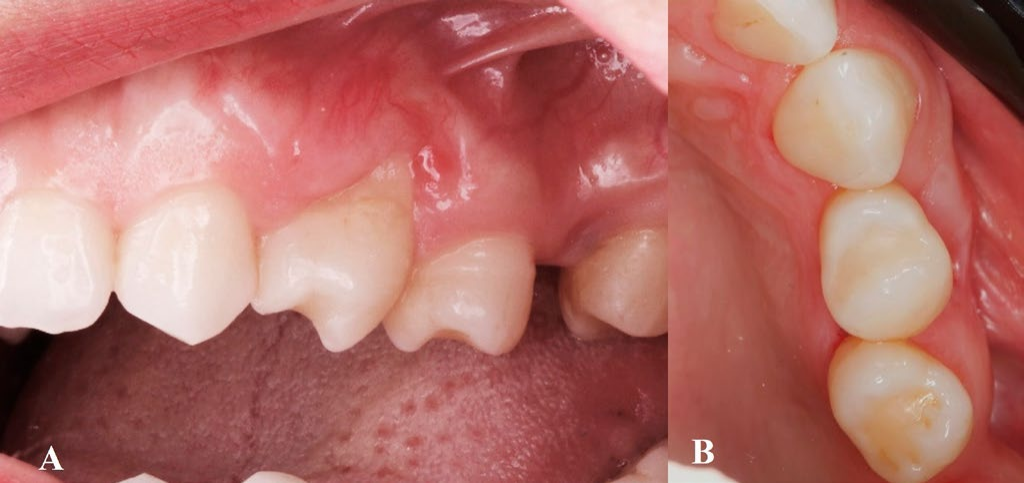
Postoperative clinical outcomes at 3 months following treatment with a laterally positioned flap combined with a connective tissue graft. A) Buccal view showing partial reduction of gingival recession depth and keratinized tissue gain. B) Occlusal view demonstrating increased gingival tissue thickness in the treated area.

**Fig. 5:**
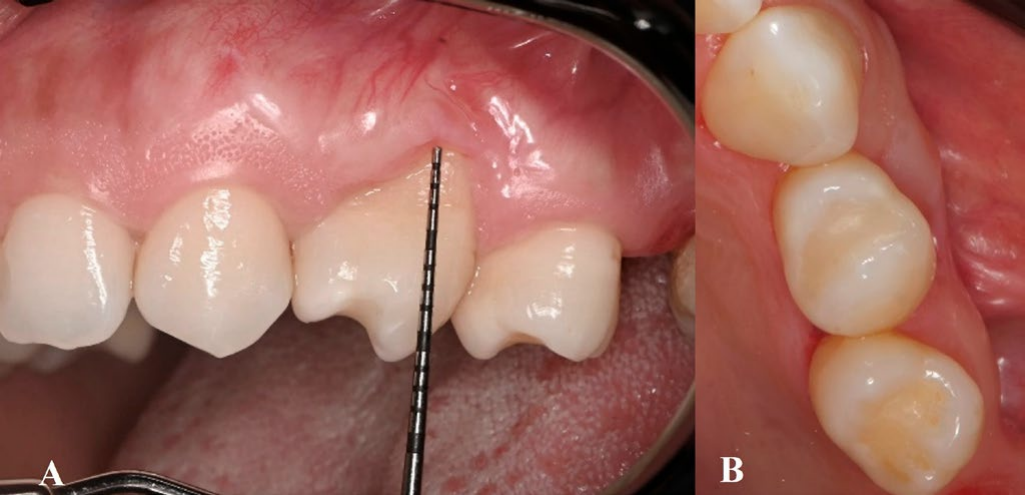
Postoperative clinical outcomes at 6 months following treatment with a laterally positioned flap combined with a connective tissue graft. A) Buccal view showing stable partial root coverage. B) Occlusal view demonstrates persistence of gingival volume and improved soft tissue contour around tooth #24.

Postoperative instructions included a soft diet for five days and the administration of ibuprofen 400 mg and paracetamol 500 mg for three days to manage discomfort. The patient was also advised to rinse with 0.12% chlorhexidine + 0.05% cetylpyridinium chloride (PerioAid Treatment; DENTAID, Spain) twice daily for 14 days to maintain plaque control. Sutures were removed after 14 days, and follow-up evaluations were conducted at 3 and 6 months postoperatively (Fig. 4 & 5).

At the 6-month follow-up, the surgical site exhibited excellent plaque control and tissue stability. Although complete root coverage was not anticipated due to the unfavorable anatomical conditions including tooth malposition, thin phenotype and absence of keratinized tissue, a significant clinical improvement was observed. Approximately 60% of the previously exposed root surface was successfully covered. In addition, 3 mm of keratinized tissue was gained at the recipient site, and a noticeable increase in gingival thickness was achieved, contributing to an improved periodontal phenotype and esthetic outcome (Fig. 4).

## DISCUSSION

The present case report describes the mucogingival management of a rotated premolar with gingival recession, thin periodontal phenotype, and absence of keratinized tissue using a laterally moved coronally advanced flap (LMCAF) in combination with a connective tissue graft. Despite the anatomical limitations, including tooth malposition, papillary involvement, and lack of keratinized tissue, favorable clinical outcomes were achieved, including partial root coverage, phenotypic modification, and gain of keratinized tissue.

Tooth malposition, particularly rotation, has been identified as a significant limiting factor for achieving complete root coverage (CRC). As reported by Zucchelli et al. ^6^. and Cairo et al. ^20^, rotated teeth typically present with a thin buccal bone plate and apically displaced CEJ-contact point relationships, which can compromise the height of the interdental papilla and the surgical endpoint for CRC. In this case, although CRC was not achieved, the 60% root coverage obtained is clinically relevant given the unfavorable conditions.

Although alternative surgical techniques might have been appropriate, the chosen approach was based on anatomical features such as tooth malposition, significant recession depth (>5mm), and limited keratinized tissue (KT) ^3^^,^^4^. The coronally advanced flap (CAF) is considered the treatment of choice for single recessions; however, it requires adequate KT in the apical area of the defect ^21^, and in this case, the presence of a shallow vestibule contraindicated its use ^19^. Tooth rotation (TR) further complicated the situation by altering the topographic relationship between the cementoenamel junction (CEJ) and the interdental papillae, resulting in a smaller soft tissue coverage area relative to the root exposure ^10^. The lateral flap was planned to increase KT prior to OT, supported by evidence from a systematic review reporting that the laterally positioned flap yields greater KT gain than CAF ^20^. An alternative surgical option could have been a free gingival graft, which effectively increases both the thickness and width of KT in a single procedure by removing muscle attachments ^22^; however, this technique may cause esthetic concerns due to color mismatch between the palatal donor site and adjacent tissues ^23^. After discussing these risks and considering the patient’s esthetic expectations, this approach was declined.

The LMCAF, originally described by Grupe and Warren ^24^, has been modified several times in order to reduce the risk of GR in the donor site. Zucchelli et al. ^19^ mixed the approach with a CAF while Staffileno ^25^ proposed the use of a partial-thickness flap to cover the root exposure. Also, Grupe and Warren ^26^ recommended a submarginal incision to preserve the marginal integrity of the adjacent tooth.

A key advantage of the LMCAF is that it avoids a second surgical incision, unlike CAF, but it requires sufficient KT lateral to the recession defect ^19^. In this case, the patient had adequate KT on the canine and interdental area between teeth 23 and 24 to allow flap mobilization. However, upon evaluating tissue thickness, a bone ridge deficiency was identified, prompting the use of a connective tissue graft (CTG) to enhance ridge contour. Subepithelial CTG increases mucosal thickness and improves the predictability of root coverage outcomes by providing KT gain ^27^.

One limitation of this technique is that it is not considered highly predictable for complete root coverage. Studies on the LPF technique have reported an overall average of 60 to 90% across all investigations ^28^^,^^29^. Moreover, there remains a risk of GR or bone loss at the donor site ^19^. 

It is important to emphasize that the primary objective was not solely esthetic improvement but rather the creation and modification of the gingival phenotype. Increasing the width of KT and GT may improve the biomechanical stability of the periodontium, providing greater resilience to functional demands and minimizing the risk of further GR during orthodontic movement ^3^^,^^9^^,^^10^. To date, there is no clear consensus in the literature regarding the optimal timing of periodontal plastic surgery in relation to orthodontic treatment, and whether such procedures should be performed before or after tooth movement remains a subject of debate ^14^^,^^27^. This report contributes clinical evidence supporting the potential benefits of a pre-orthodontic mucogingival approach in high-risk cases. However, the partial root coverage achieved in this case highlights the value of an interdisciplinary therapeutic approach involving multiple mucogingival surgical phases. 

From a prognostic perspective, this case shows a favorable short-term outlook, with stable soft tissues, increased KT, and adequate GT at six months. In the medium term, the phenotype modification is expected to improve periodontal stability and reduce the likelihood of GR progression ^3^^,^^9^^,^^10^. Nonetheless, OT in patients with a history of GR carries an inherent risk of recurrence, particularly with excessive buccal movement or uncontrolled forces ^14^^,^^27^. This risk can be minimized through careful orthodontic planning, the application of light, controlled forces, continuous periodontal monitoring ^5^^,^^22^, meticulous oral hygiene, and regular maintenance visits to detect and manage early signs of inflammation or recession

Clinically, this case is relevant because it addresses a deep buccal GR (≥ 5 mm) in a rotated tooth with thin phenotype and absence of KT-conditions that significantly limit surgical predictability ^9^^,^^16^^,^^20^. By treating these factors before orthodontic movement, the clinician can improve gingival margin stability, preserve periodontal health, and enhance long-term esthetic outcomes ^8^^,^^23^. The interdisciplinary strategy followed here underscores the importance of integrating periodontal phenotype modification into orthodontic treatment planning to optimize results and reduce complications ^22^^,^^27^.

## CONCLUSION

The management of deep gingival recession in a rotated tooth presents significant clinical challenges due to altered anatomical relationships and limited keratinized tissue. This case demonstrates that a laterally moved coronally advanced flap combined with a connective tissue graft can effectively increase mucosal thickness and keratinized tissue width, resulting in stable partial root coverage. Such mucogingival preparation prior to orthodontic treatment may enhance periodontal stability and improve treatment prognosis. An individualized, anatomy-driven approach is essential for optimizing outcomes in complex recession defects, emphasizing the importance of interdisciplinary care in periodontal and orthodontic management.

## DECLARATIONS

Consent to participate. Written informed consent was obtained from patient to authorize the research.

Consent to publish. Informed consent was obtained from the participant.
